# Safety and efficacy of tumour-treating fields (TTFields) therapy for newly diagnosed glioblastoma in Japanese patients using the Novo-TTF System: a prospective post-approval study

**DOI:** 10.1093/jjco/hyad001

**Published:** 2023-01-16

**Authors:** Ryo Nishikawa, Fumiyuki Yamasaki, Yoshiki Arakawa, Yoshihiro Muragaki, Yoshitaka Narita, Shota Tanaka, Shigeru Yamaguchi, Akitake Mukasa, Masayuki Kanamori

**Affiliations:** Department of Neuro-Oncology/Neurosurgery, Saitama Medical University International Medical Center, Saitama, Japan; Department of Neurosurgery, Hiroshima University Hospital, Hiroshima, Japan; Department of Neurosurgery, Kyoto University Graduate School of Medicine, Kyoto, Japan; Department of Neurosurgery, Tokyo Women’s Medical University Hospital, Tokyo, Japan; Department of Neurosurgery and Neuro-Oncology, National Cancer Center Hospital, Tokyo, Japan; Department of Neurosurgery, The University of Tokyo Hospital, Tokyo, Japan; Department of Neurosurgery, Hokkaido University Hospital, Sapporo, Japan; Department of Neurosurgery, Kumamoto University Hospital, Kumamoto, Japan; Department of Neurosurgery, Tohoku University Hospital, Sendai, Japan

**Keywords:** skin, new technology/instruments, interventional therapy, CNS

## Abstract

**Background:**

Tumour-treating fields therapy is a locoregional, anti-cancer treatment. Efficacy and safety of tumour-treating fields therapy in adults with newly diagnosed glioblastoma were demonstrated in the pivotal phase 3 EF-14 study (NCT00916409). Here, we report post-approval data of tumour-treating fields therapy in Japanese patients with newly diagnosed glioblastoma.

**Methods:**

Unsolicited post-marketing surveillance data from Japanese patients with newly diagnosed glioblastoma treated with tumour-treating fields therapy (December 2016–June 2020) were retrospectively analysed. The primary endpoints were skin, neurological and psychiatric adverse events. The secondary endpoints were 1- and 2-year overall survival rates, and the 6-month progression-free survival. adverse events were analysed using MedDRA v24.0. The overall survival and progression-free survival were assessed using the Kaplan–Meier survival analysis (log-rank testing). The Cox proportional hazard regression analyses were also performed.

**Results:**

Forty patients with newly diagnosed glioblastoma were enrolled (62.5% male; median age 59 years; median baseline Karnofsky Performance Scale score 90). The most common tumour-treating-fields-therapy-related adverse event was beneath-array local skin reaction (60% of patients). The adverse events were mostly mild to moderate in severity. Neurological disorders were observed in 2.5% patients (one patient reported dysesthesia). No psychiatric disorders were reported. The 1- and 2-year overall survival rates were 77.9% (95% CI 60.6–88.3) and 53.6% (35.5–68.7%), respectively. The 6-month progression-free survival was 77.5% (61.2–87.6%). These survival rates compare favourably with those in the EF-14 trial (1- and 2-year overall survival rates: 73% [69–77%] and 43% [39–48%], respectively; 6-month progression-free survival rate: 56% (51–61%).

**Conclusion:**

This post-approval, real-world evidence study revealed no new safety signals and suggests the safety and efficacy of tumour-treating fields therapy in Japanese patients with newly diagnosed glioblastoma.

## Teaser

This post-approval, real-world study revealed no new safety signals and is suggestive of the safety and efficacy of tumour-treating fields therapy in Japanese patients with newly diagnosed glioblastoma.

## Introduction

Glioblastoma (GBM) is the most common malignant primary brain tumour, accounting for 12% of all the primary brain tumours in Japan (1), and with an estimated global incidence rate of 3.2 per 100 000 individuals (2,3). GBM is associated with a very poor prognosis, with an estimated 5-year survival rate of 15.5% in neurosurgical cases (1). Regardless of the treatment, GBM invariably recurs within a median time interval of <7 months and treatment options for the management of recurrences are lacking (2).

The Society for Neuro-Oncology (SNO), the European Association of Neuro-Oncology (EANO), the Japan Society of Clinical Oncology (JSCO), the Japan Neurosurgical Society (JNS) and the Japan Society for Neuro-Oncology (JSNO) recommend that patients with newly diagnosed (nd) GBM undergo maximal safe tumour resection, followed by radiation therapy with concomitant and then adjuvant temozolomide (TMZ) chemotherapy (2,4). However, the addition of Tumor Treating Fields therapy (TTFields; Optune®, Novocure® GmbH, Device Manufacturer) to maintenance TMZ for ndGBM has now been incorporated into this standard of care (SOC) following approval in China, EU, Japan and USA, (5,6).

TTFields are low intensity, intermediate frequency (150–200 kHz), alternating electric fields that cause mitotic arrest and apoptosis leading to the suppression of tumour growth and spread (7–13). TTFields have also been shown to exert biophysical forces on a variety of charged and polarizable molecules, impacting DNA repair, autophagy, cancer cell migration, membrane permeability and immunological response processes, in addition to their antimitotic effects (7,8,14–17). TTFields therapy is locoregional, rather than systemic, since the electric fields are continuously generated by a wearable device, and delivered via arrays placed directly on the surface of the scalp at the site of the tumour.

TTFields therapy is approved for the treatment of adult patients with malignant pleural mesothelioma, ndGBM and recurrent GBM (5,6,18), based on efficacy and safety data from the STELLAR, EF-14 and EF-11 clinical studies, respectively (19–23).

The pivotal phase 3 EF-14 clinical study (NCT00916409), which included patients with ndGBM whose tumour was resected or biopsied and who had completed concomitant radiochemotherapy, compared subsequent TTFields therapy with TMZ versus maintenance TMZ alone (22). Compared with the patients receiving TMZ only, the patients receiving TTFields therapy had significant improvements in progression-free survival (PFS) (6.7 vs. 4.0 months [hazard ratio (HR), 0.63; 95% confidence interval (CI) 0.52–0.76; *P* < 0.001]) and overall survival (OS) (20.9 vs. 16.0 months [HR, 0.63; 95% CI 0.53–0.76; *P* < 0.001]). Furthermore, a post hoc analysis of the EF-14 data reported that continuation of TTFields therapy after the first progression significantly improved OS (24). Survival benefits were also evident among a subgroup of vulnerable elderly patients (25).

The addition of TTFields therapy to TMZ did not significantly increase the rates of systemic adverse events (AEs), and the safety profile of the two treatment groups were comparable, with the exception of a higher incidence of localized skin reactions among the patients receiving concomitant TTFields therapy (22). An absence of systemic AEs was also observed in the pivotal EF-11 study (NCT00379470) in patients with recurrent GBM (23). In this study, TTFields monotherapy was comparable in efficacy with the best available treatment according to the physician’s choice, but displayed a more favourable safety profile. The patients experienced fewer severe AEs, and the AEs were generally localized to the scalp rather than the systemic events typically associated with chemotherapy. Advice on the prevention and management of dermatologic scalp AEs, which are known to be associated with TTFields therapy, is available (26,27).

Real-world evidence from a global surveillance study of >10 000 patients with GBM (28) confirmed the favourable safety profile of TTFields therapy in GBM reported in the pivotal EF-11 and EF-14 studies. Overall, the AEs were mainly localized to the treatment area and the paediatric patients experienced similar rates of skin irritation as the adult and elderly patients. There were also no increases in systemic effects or the new safety concerns (28).

This paper reports additional real-world safety and efficacy data for TTFields therapy in Japanese patients with ndGBM (EF-29).

## Methods

### Study design and patient population

Unsolicited post-marketing surveillance data from Japanese patients with ndGBM who received TTFields therapy (December 2016–June 2020) were retrospectively analysed. Patients with histologically confirmed supratentorial ndGBM were eligible for inclusion. Exclusion criteria were as follows: patients treated outside of the contract or enrollment periods, patients with a history of treatment with the relevant NovoTTF device before enrollment, patients who did not give or withdrew consent and those who were lost to follow-up.

### Endpoints

The primary endpoints were the rate of the following AEs: skin irritations, neurological and psychiatric disorders.

The secondary endpoints included assessment of 1- and 2-year OS and 6-month PFS rates. The exploratory endpoints included the analysis of the following factors potentially influencing survival: patient age, surgical resection method and baseline Karnofsky Performance Scale (KPS) score.

### Safety analysis

The rates of AEs, patient information and device usage data were collected and analysed. All the events entered in the ‘AE report’ field in the paper case report form were subjected to safety assessments, in accordance with an internal standard operating procedure. In the case of queries surrounding the entry of events in areas other than the AE report field, follow-up investigations were conducted with the physician who made the entry and data clarified accordingly. The AEs that occurred after the discontinuation of the device, and which had been assessed as not causally related by the physician, were excluded from the analysis. AEs were named according to the Medical Dictionary for Regulatory Activities (MedDRA) version 24.0 preferred terms. AEs were classed as mild, moderate, severe, life-threatening and fatal, and defined as related, not related or unknown by the treating physician. Serious AEs were based on the ICH International Pharmaceutical Glossary (MedDRA/J) version 24.0 Classification.

### Efficacy analysis

Disease progression was assessed every 2 months. Where a magnetic resonance imaging scan was available, disease progression was determined according to the Response Assessment in Neuro-Oncology criteria.

### Statistical analysis

A minimum of 40 patients was calculated as necessary to detect one case of anxiety (a rate of incidence 33/437) with a power of 95%, and this was therefore considered to be the required minimum sample size for analysis.

Survival rates (OS and PFS) were estimated using the Kaplan–Meier method. OS of the following subgroups was assessed (potential differences were investigated using a log-rank test): sex; age; treatment history of GBM; surgical resection method (biopsy, partial resection and gross total resection); KPS score ≥70 or <70 at baseline; average daily treatment time ≥18 h or <18 h. Descriptive statistics of the AEs (occurrence rate) was conducted as part of the primary analysis endpoint.

The Cox proportional hazard regression model was used to analyse the impact of the following covariates on prognosis: age degree of surgical resection (biopsy, partial resection and gross total resection) KPS. Statistical significance at *P* < 0.05 was determined using the chi-squared test. In addition, the survival rates were analysed by the Cox proportional hazard regression analyses using the Wald chi-square test. The analyses aims to examine whether the baseline characteristics: age, degree of surgical resection (biopsy, partial resection and gross total resection) and baseline KPS are prognostic factors.

## Results

### Study population

In total, retrospective data from 40 patients, across nine Japanese sites, were collected and evaluated (Fig. S1). Baseline characteristics are summarized in Table 1. The majority (62.5%) of patients were male; median age was 59 years, with 60.0% of patients <65 years of age, and median KPS score was 90.

**Table 1:** Baseline characteristics

	Total (*N* = 40)
Age, (years), median (range)	59 (19–75)
Age, (years), *n* (%)	
<65	24 (60)
≥65	11 (27.5)
Unknown	5 (12.5)
Male, *n* (%)	25 (62.5)
KPS score, median (range)	90 (60–100)
60, *n* (%)	1 (2.5)
70, *n* (%)	4 (10.0)
80, *n* (%)	8 (20.0)
90, *n* (%)	17 (42.5)
100, *n* (%)	9 (22.5)
Unknown, *n* (%)	1 (2.5)
Concomitant steroids, *n* (%)	
Yes	32 (80.0)
No	7 (17.5)
Unknown	1 (2.5)
History of GBM resection, *n* (%)	
Biopsy	3 (7.5)
Partial	12 (30.0)
Gross total	23 (57.5)
Unknown/data not provided	2 (5.0)

GBM, glioblastoma; KPS, Karnofsky Performance Score.

**Table 2:** Adverse events and association with TTFields therapy

	TTFields-therapy-related (Y/N)	Total (*N* = 40)
Number of reported AEs, events	–	31
Patients with ≥1 AE, *n* (%)	–	24 (60.0)
Skin-related AEs, *n* (%)		
Dermatitis	Y	10 (25.0)
Itching	Y	5 (12.5)
Erythema	Y	3 (7.5)
Eczema	Y	2 (5.0)
Skin erosion	Y	2 (5.0)
Blisters	Y	1 (2.5)
Rash	Y	1 (2.5)
Skin disorder	Y	1 (2.5)
Skin pain	Y	1 (2.5)
Skin ulcer	Y	1 (2.5)
Neurological AEs, *n* (%)		
Dysesthesia	N	1 (2.5)
Other, n (%)		
Malignant neoplasm progression	N	1 (2.5)
Discomfort at site of medical device use	Y	1 (2.5)
Skin injury	Y	1 (2.5)

AEs, adverse events; N, no; TTFields, Tumor Treating Fields; Y, yes.

**Figure 1:**
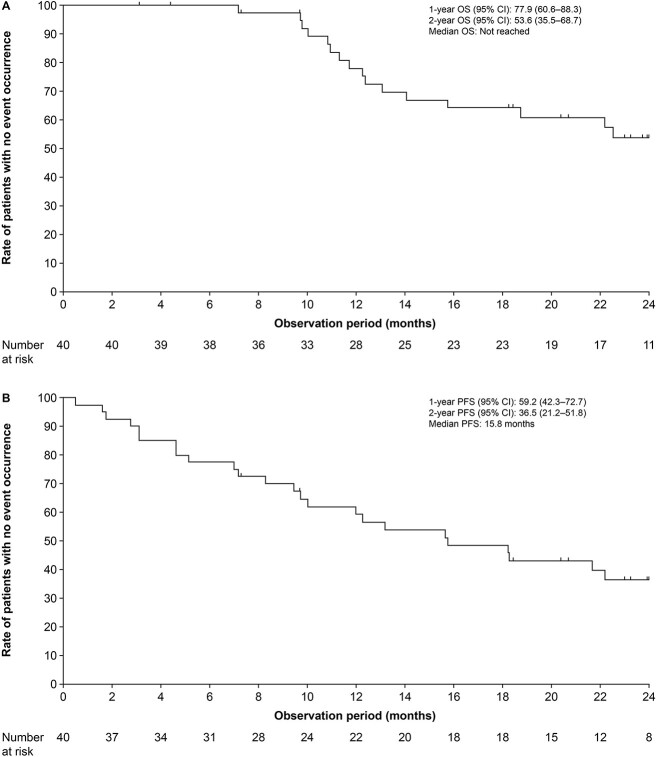
Kaplan–Meier curves of (A) overall survival and (B) progression-free survival.

### Safety analysis

Overall, 24 (60%) patients reported ≥1 AE. All these patients experienced skin irritation reactions that were generally mild-to-moderate in severity. Most were localized skin irritations with dermatitis being the most frequently reported (25%) (Table 2). In 21 patients (53%), skin-related AEs occurred within 6 months of starting treatment. Treatment was permanently stopped in one patient and temporarily stopped in three patients as a result of skin AEs; all four patients experienced resolution. One patient experienced dysesthesia within 12 months of starting treatment; this was not considered to be a serious neurological event. No psychiatric AEs were reported. Other AEs reported were malignant neoplasm progression, discomfort at site of medical device and skin injury, all of which were reported by one patient each. TTFields-therapy-related AEs comprised of skin irritation symptoms, skin-related injury and discomfort at the treatment site (Table 2). In total, 10 patients experienced serious AEs (15 events in total); none were related to TTFields therapy (Table 3).

**Table 3:** Incidence of serious adverse events

Serious adverse events, *n* (%)	Total (*N* = 40)
Malignant neoplasm progression	6 (15.0)
Vertebral compression fracture	2 (5.0)
Epilepsy	1 (2.5)
Monoplegia	1 (2.5)
Seizure	1 (2.5)
Cholecystitis	1 (2.5)
Gait disturbance	1 (2.5)
Lymphocyte decrease	1 (2.5)
Wrist fracture	1 (2.5)

### Survival analysis

One and 2-year OS rates were 77.9% (95% CI 60.6–88.3) and 53.6% (95% CI 35.5–68.7), respectively (Fig. 1A). Six-month PFS rate was 77.5% (95% CI 61.2–87.6) (Fig. 1B).

**Figure 2:**
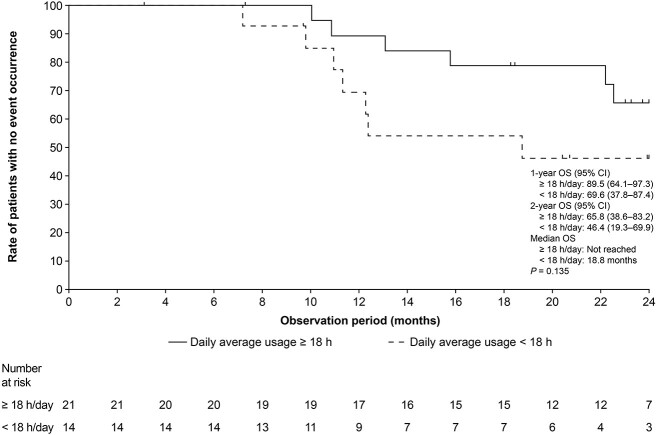
Kaplan–Meier curve of overall survival, according to time on TTFields therapy. TTFields, Tumor Treating Fields.

The 2-year OS rate in patients who used TTFields therapy for an average of ≥18 h/day during the duration of the contract was 65.8% (95% CI 38.6–83.2), demonstrating a trend towards increased survival relative to patients who used TTFields therapy for an average of <18 h/day (46.4% [95% CI 19.3–69.9]), *P* = 0.14 (Fig. 2).

As age was unknown for five patients, the Cox proportional hazard regression analysis was performed using the survival data from 35 patients. Analyses of OS using the Cox proportional hazard modelling found survival to be significantly improved relative to each year decrease in age (univariate HR: 1.050 [95% CI 1.002–1.100], *P* = 0.040; multivariate HR: 1.053, [95% CI 1.003–1.105] *P* = 0.037; Table S1). Degree of surgical resection and baseline KPS score were not significant prognostic factors for OS (Table S1). Likewise, age, degree of surgical resection and baseline KPS score were not significantly associated with PFS (all *P* > 0.05) (Table S2).

## Discussion

This post-marketing surveillance study in Japanese patients adds to the growing body of real-world safety data for TTFields therapy in patients with ndGBM. The study revealed no new safety signals; most events were mild-to-moderate, localized skin irritations associated with TTFields therapy.

Results are in line with previous studies which have also shown skin reactions to be the most common TTFields-therapy-related AEs. In the pivotal phase 3 EF-14 study, 52% of patients experienced mild-to-moderate skin irritation (22), while in the large-scale global post-marketing surveillance data analysis, 38% of patients with ndGBM experienced skin reactions with TTFields therapy (28). Similar high rates of occurrence of skin AEs have been reported for the TTFields-therapy-treated patients with recurrent GBM (28,29). Skin-related AEs can be managed by early prophylactic interventions, such as optimal shaving and periodic shifting of the array position, and good patient management strategies; for example, use of topical corticosteroids or antibiotics (5,27). In EF-14, the AEs associated with concomitant TMZ (e.g. leukopenia or lymphopenia) were reported and were within the expected levels (22). However, the AEs associated with any concurrent anti-cancer treatments were not captured in this study, likely due to the retrospective design and potential reporting bias towards the TTFields-therapy-related AEs of the treating physicians.

Of note, while the proportion of GBM patients ≥65 years of age has been reported to be 45% in the Japanese brain tumour registry (1), only 28% (11/40) of patients were ≥65 years of age in this study. The slightly younger population in this study may potentially reflect differences in patient and/or healthcare professional drivers and barriers to initiating TTFields therapy for younger versus older patients. The male:female ratio of patients receiving TTFields therapy in this study (62.5% male) was slightly higher than the GBM patient population as a whole in Japan (58% male) (1), but in line with that previously reported in the global TTFields therapy post-marketing study (66.3% male) (28).

Although the median KPS was consistent across this study and EF-14, the median age of patients in this study was slightly higher than in EF-14 (59.0 [19–75] years vs. 56.0 [19–83] years, respectively). Despite this, survival data in this study compared favourably with rates observed in the EF-14 study population. In EF-14, 73% and 43% of the TTFields-therapy-treated patients were alive after 1 and 2 years, respectively, with 56% experiencing PFS for 6 months (22).

In this study, a trend of an increased 2-year OS was seen in patients who used TTFields therapy for ≥18 h/day compared with those who used it for <18 h/day. This is consistent with the previous observations that TTFields therapy with a maximal monthly compliance rate ≥75% (≥ 18 h/day) corresponds to greater survival benefit in patients with GBM (2,3,29–31).

The retrospective study design and small sample number represent limitations of the study, as the analyses could not be statistically powered. The lack of a control arm means that there was no way of comparing TTFields therapy with other treatment strategies. Additionally, the study did not include reporting on concurrent therapies, such as steroids, or other treatments such as bevacizumab. As such, the impact of these treatments on safety outcomes cannot be adjusted for, and this should therefore be taken into account when evaluating the data, as concurrent therapies may have potentially affected the AE incidences, for example the use of topical steroids may have affected skin fragility, impacting the placement of arrays. Furthermore, as data were collected retrospectively, reporting of AEs may not have been carried out consistently.

This analysis provides evidence that TTFields therapy is well tolerated in Japanese patients, with survival rates comparable with those observed in the pivotal phase 3 EF-14 study. Although these data are suggestive of efficacy in a Japanese population, results should be interpreted with caution, due to the limitations described above.

## Conclusion

TTFields therapy was generally well tolerated, with no new safety signals in Japanese patients with ndGBM, despite the population having a high burden of disease. The AE profile was comparable with published clinical trial data and real-world evidence, with localized skin reactions being the most frequently reported TTFields-therapy-associated AEs.

In this post-marketing study, the younger patients with better performance scores tended to be included, in contrast to a real-world population in which patients with ndGBM would typically be older with a less favourable KPS score. Even so, data reported here are suggestive of the safety and efficacy of TTFields therapy in Japanese patients with ndGBM, and support use in this patient population.

## Supplementary Material

EF_29_manuscript_Suppl_Material_18July2022_hyad001

## Data sharing

The datasets generated during and/or analysed during this study are available for 3 years after the date of publication. Please contact Piet Hinoul, (phinoul@novocure.com) for inquiries about access.
