# Correction to: Safety and efficacy of Tumor Treating fields (TTFields) therapy for newly diagnosed glioblastoma in Japanese patients using the Novo-TTF System: a prospective post-approval study

**DOI:** 10.1093/jjco/hyad014

**Published:** 2023-02-22

This is a correction to: Ryo Nishikawa, Fumiyuki Yamasaki, Yoshiki Arakawa, Yoshihiro Muragaki, Yoshitaka Narita, Shota Tanaka, Shigeru Yamaguchi, Akitake Mukasa, Masayuki Kanamori, Safety and efficacy of Tumor Treating Fields (TTFields) therapy for newly diagnosed glioblastoma in Japanese patients using the Novo-TTF System: a prospective post-approval study, Japanese Journal of Clinical Oncology, 2023;, hyad001, https://doi.org/10.1093/jjco/hyad001

The originally published version of this manuscript contained several errors, which occurred due to publisher oversight.

‘Tumor Treating Fields (TTFields) therapy’ was incorrectly ‘written’ as ‘tumour-treating fields (TTFields) therapy’ throughout the manuscript.

‘Novo TTF device’ was incorrectly written as ‘NovoTT device’ in the Methods section.

In the following sentence, ‘(Table S1)’ should have been given as ‘(Fig. S1)’:

In total, retrospective data from 40 patients, across nine Japanese sites, were collected and evaluated (Table S1).

The following sentence contained the incorrect P value:

Analyses of OS using the Cox proportional hazard modelling found survival to be significantly improved relative to each year decrease in age (univariate HR: 1.050 [95% CI 1.002–1.100], P = 0.04; multivariate HR: 1.053, [95% CI 1.003–1.105] P = 0.04; Table S1).

The sentence now reads as follows:

Analyses of OS using the Cox proportional hazard modelling found survival to be significantly improved relative to each year decrease in age (univariate HR: 1.050 [95% CI 1.002–1.100], P = 0.040; multivariate HR: 1.053, [95% CI 1.003–1.105] P = 0.037; Table S1).

In the below sentence, there was an error in the name of the referenced study, whereby ‘E-14’ should have been given as ‘F-14’:

This analysis provides evidence that TTFields therapy is well tolerated in Japanese patients, with survival rates comparable with those observed in the pivotal phase 3 E-14 study.

The link to Good Publication Practice guidelines, https://www.acpjournals.org/doi/10.7326/M22-1460, was omitted from the following sentence:

Writing and editorial support provided by Prime was funded by Novocure Inc. and conducted according to the Good Publication Practice guidelines (link).

Finally, a number of minor corrections were required to references.

These errors have now been corrected; the Publisher apologizes for not correcting them at an earlier production stage.

