# Supplementary material for: Safety and efficacy of tumour-treating fields (TTFields) therapy for newly diagnosed glioblastoma in Japanese patients using the Novo-TTF System: a prospective post-approval study
Source: Jpn J Clin Oncol. 2023 Jan 16;53(5):371–7. doi: 10.1093/jjco/hyad001 (PMC10150168; doi:10.1093/jjco/hyad001)
Supplement: EF_29_manuscript_Suppl_Material_18July2022_hyad001 [file ef_29_manuscript_suppl_material_18july2022_hyad001.docx]

# Supplemental Information

Table S1. Cox proportional hazard regression model for overall survival

|  | **Univariate analysis** | | **Multivariate analysis** | |
| --- | --- | --- | --- | --- |
| **Factor** | **HR (95% CI)** | ***P* value** | **HR (95% CI)** | ***P* value** |
| Age (years) | 1.050 (1.002, 1.100) | **0.040*** | 1.053 (1.003, 1.105) | **0.037*** |
| GBM resection |  |  |  |  |
| Biopsy | – | – | – | – |
| Partial | 1.191 (0.143, 9.914) | 0.872 | 0.339 (0.035, 3.310) | 0.352 |
| Gross total | 0.893 (0.113, 7.073) | 0.915 | 0.261 (0.029, 2.340) | 0.230 |
| Baseline KPS score | 1.007 (0.960, 1.056) | 0.785 | 1.017 (0.963, 1.074) | 0.547 |

**P* < 0.05 on chi-square test.
CI, confidence interval; GBM, glioblastoma; HR, hazard ratio; KPS, Karnofsky Performance Scale.

Table S2. Cox proportional hazard regression model for progression-free survival

|  | **Univariate analysis** | | **Multivariate analysis** | |
| --- | --- | --- | --- | --- |
| **Factor** | **HR (95% CI)** | ***P* value** | **HR (95% CI)** | ***P* value** |
| Age (years) | 1.020 (0.988, 1.053) | 0.225 | 1.024 (0.990, 1.059) | 0.168 |
| GBM resection |  |  |  |  |
| Biopsy | – | – | – | – |
| Partial | 2.585 (0.327, 20.438) | 0.368 | 1.077 (0.130, 8.904) | 0.945 |
| Gross total | 1.897 (0.249, 14.446) | 0.537 | 0.814 (0.104, 6.378) | 0.845 |
| Baseline KPS score | 1.012 (0.971, 1.055) | 0.576 | 1.015 (0.968, 1.064) | 0.534 |

CI, confidence interval; GBM, glioblastoma; HR, hazard ratio; KPS, Karnofsky Performance Scale.
